# *Candida albicans* Hypha Formation and Mannan Masking of β-Glucan Inhibit Macrophage Phagosome Maturation

**DOI:** 10.1128/mBio.01874-14

**Published:** 2014-12-02

**Authors:** Judith M. Bain, Johanna Louw, Leanne E. Lewis, Blessing Okai, Catriona A. Walls, Elizabeth R. Ballou, Louise A. Walker, Delyth Reid, Carol A. Munro, Alistair J. P. Brown, Gordon D. Brown, Neil A. R. Gow, Lars P. Erwig

**Affiliations:** Aberdeen Fungal Group, University of Aberdeen, Aberdeen, United Kingdom

## Abstract

*Candida albicans* is a major life-threatening human fungal pathogen in the immunocompromised host. Host defense against systemic *Candida* infection relies heavily on the capacity of professional phagocytes of the innate immune system to ingest and destroy fungal cells. A number of pathogens, including *C. albicans*, have evolved mechanisms that attenuate the efficiency of phagosome-mediated inactivation, promoting their survival and replication within the host. Here we visualize host-pathogen interactions using live-cell imaging and show that viable, but not heat- or UV-killed *C. albicans* cells profoundly delay phagosome maturation in macrophage cell lines and primary macrophages. The ability of *C. albicans* to delay phagosome maturation is dependent on cell wall composition and fungal morphology. Loss of cell wall *O*-mannan is associated with enhanced acquisition of phagosome maturation markers, distinct changes in Rab GTPase acquisition by the maturing phagosome, impaired hyphal growth within macrophage phagosomes, profound changes in macrophage actin dynamics, and ultimately a reduced ability of fungal cells to escape from macrophage phagosomes. The loss of cell wall *O*-mannan leads to exposure of β-glucan in the inner cell wall, facilitating recognition by Dectin-1, which is associated with enhanced phagosome maturation.

## IMPORTANCE

Innate cells engulf and destroy invading organisms by phagocytosis, which is essential for the elimination of fungal cells to protect against systemic life-threatening infections. Yet comparatively little is known about what controls the maturation of phagosomes following ingestion of fungal cells. We used live-cell microscopy and fluorescent protein reporter macrophages to understand how *C. albicans* viability, filamentous growth, and cell wall composition affect phagosome maturation and the survival of the pathogen within host macrophages. We have demonstrated that cell wall glycosylation and yeast-hypha morphogenesis are required for disruption of host processes that function to inactivate pathogens, leading to survival and escape of this fungal pathogen from within host phagocytes. The methods employed here are applicable to study interactions of other pathogens with phagocytic cells to dissect how specific microbial features impact different stages of phagosome maturation and the survival of the pathogen or host.

## INTRODUCTION

*Candida* species represent the fourth most frequent cause of bloodstream infection in hospitalized patients, with mortality in 40% of cases, even when antifungal therapy is administered (1). Of these infections, *Candida albicans*, characterized by its ability to form parallel-sided hyphae that invade epithelia and puncture or resist killing by phagocytes, is the most frequent causative agent. *Candida* species are constituents of healthy human gastrointestinal mucosal microflora and may be present in up to 80% of the population; therefore, opportunistic infections seeded from a commensal reservoir can arise following breach of normal defenses or perturbations in immune or microbiological homeostasis (2). The capacity of professional phagocytes, including neutrophils and macrophages, to ingest and destroy invading fungal cells underpins the sentinel activity of the innate immune response upon host invasion. However, comparatively little is known about the fungus-associated factors that control maturation of macrophage phagosomes following phagocytosis of fungal cells. This knowledge gap is addressed in this study, in which we demonstrate that *C. albicans* hyphae and the polysaccharides of the outer cell wall disrupt progression of phagosome maturation.

Phagocytes deliver pathogens into the phagosome, an organelle that matures by sequential interactions with endocytic and lysosomal compartments. The process is regulated by Rab GTPases which coordinate vesicular traffic to phagosomes (3). Maturation remodels the phagosomal membrane and lumenal content, promoting acquisition of vacuolar ATPase (v-ATPase) to pump protons inwardly to a progressively acidified lumen (4). Defensins and the generation of reactive oxygen and nitrogen species also contribute to a cytotoxic environment within phagosomes (5). Fusion of lysosomes then delivers hydrolytic enzymes, including lipases and proteases, such as cathepsins, which function optimally at low pH (6). The digestion products generated are then presented on major histocompatibility complex (MHC) class II molecules to drive adaptive immune responses in the host (7, 8). Therefore, efficient phagosome maturation is a key process in the control of infectious disease and is pivotal to both innate and adaptive immunity.

Some pathogens have evolved mechanisms to avoid phagosome-mediated inactivation, to promote their survival and replication within the host. These include eubacteria (*Mycobacterium tuberculosis*, *Listeria monocytogenes*, *Coxiella burnetii*, *Brucella* species, *Salmonella enterica* serovar Typhimurium, *Helicobacter pylori*, *Shigella flexneri*, *Chlamydia* species, *Legionella pneumophila*, *Francisella tularensis*, and *Rhodococcus equi*), protozoa (*Leishmania donovani*, *Trypanosoma* species, and *Toxoplasma gondii*), and fungi (*Histoplasma capsulatum*, *Candida glabrata*, and *C. albicans*) (9–19). However, the properties of fungal cells that influence the ordered series of events that occur during phagosome maturation have not been elucidated.

Previous work demonstrated that live *C. albicans* cells affect the acquisition or retention of markers indicative of alterations in the stage-specific development of lysosomal compartments (19, 20). However, the conclusions drawn from studies of fixed cells at fixed time points do not adequately reveal the temporal dynamics of phagosome maturation, particularly with respect to transient events. We have investigated the temporal dynamics of phagosome maturation in macrophages following the engulfment of *C. albicans* as a model fungal pathogen and show by live-cell imaging that fungal morphology and cell wall components critically affect these processes.

One of the most potent virulence determinants of *C. albicans* is its morphogenetic plasticity: yeast cells, pseudohyphae, and hyphae manifest in tissues depending on environmental cues and morphogens, including ambient pH, CO_2_, temperature, serum, and other micronutrients (21). Upon internalization into the macrophage phagosome, *C. albicans* is exposed to an acidic intraphagosomal environment but is able to neutralize this compartment by extrusion of ammonia (22), leading to transcriptional reprogramming of phagocytosed *C. albicans* that promotes hyphal morphogenesis (23).

We and others have demonstrated that hyphal extension is a key factor promoting fungal escape from phagocytes (24–26). We previously investigated in detail the dynamics of macrophage migration, recognition, and engulfment of *C. albicans* and found that hyphal morphotypes delay the rate of engulfment, with the geometry of filament in relation to phagocyte a contributing factor to the efficiency of phagocytic uptake (27). The same study revealed differential phagocytic recognition and uptake of *C. albicans* cell wall mutants (27).

The cell wall of *C. albicans* comprises an inner scaffold of β-1,3-glucan linked to chitin and β-1,6-glucan linked to cell wall proteins (CWPs) that are enriched in the outer cell wall (28). CWPs are predominantly linked via glycosylphosphatidylinositol (GPI) remnants to β-1,6-glucan and form the external fibrillar layer of the cell wall. They are extensively glycosylated by *N*-linked phosphomannosyl residues and short-chain *O*-linked mannans on the Ser/Thr-rich domains of the stem regions of CWP that present the globular domains of GPI proteins to the outer surface (29). The mannans of these proteins dominate the outer cell wall surface, covering and protecting the glucan-chitin skeleton. Different components of fungal cell walls are detected as pathogen-associated molecular patterns (PAMPs) via host cell pattern recognition receptors (PRRs) (30).

One of the most important PAMP-PRR engagements for fungal immunity is the recognition of β-glucan by Dectin-1, which initiates phagocytic uptake of fungi and induces a protective inflammatory Th17 response (31, 32). Intact *C. albicans* yeast cells expose β-1,3-glucan only at bud scars without mannan shielding (33), although growth *in vivo*, exposure to echinocandin antibiotics, and hyphal growth under certain cell culture conditions elicit uncloaking of inner wall β-glucan (34, 35). The arrangement of cell wall components and degree of PAMP exposure may exhibit plasticity, dependent upon the host niche, which could differentially impact immune recognition. In our previous study, we investigated the effects of glycosylation on immune recognition. We observed that a Δ*mnt1* Δ*mnt2*/Δ*mnt1* Δ*mnt2* (here referred to as the Δ*mnt1*/*2* mutant) *O*-glycosylation mutant of *C. albicans*, which *O*-mannosylates proteins with only a single mannose residue, was significantly affected in its interaction with macrophages (36, 37). This strain elicited enhanced macrophage migration, although engulfment of bound mutant cells was prolonged (27). In spite of this delay, the Δ*mnt1*/*2* mutant was taken up by macrophages in greater numbers but was less able to kill phagocytes (24, 27). Furthermore, this mutant is reported to be defective in biofilm formation, has altered adhesive properties, and was less virulent *in vivo* (36, 38). We therefore hypothesized that *O*-mannosylation and morphology influence the postengulfment processes of phagosome maturation. Here we dissect macrophage phagosome developments occurring minute by minute, following uptake of *C. albicans* by using a live-cell imaging system combined with markers of vesicular traffic and maturation of phagosomes. The data presented herewith demonstrate that phagosome maturation in macrophages is profoundly influenced by three key determinants of *C. albicans*: fungal viability, hyphal morphogenesis, and *O*-glycosylation status. These observations demonstrate that the nature of the cell wall surface dictates the efficiency of pathogen processing by phagocytes of the innate immune system.

## RESULTS

### Viable *C. albicans* cells delay phagosome maturation in macrophages.

**FIG 1:**
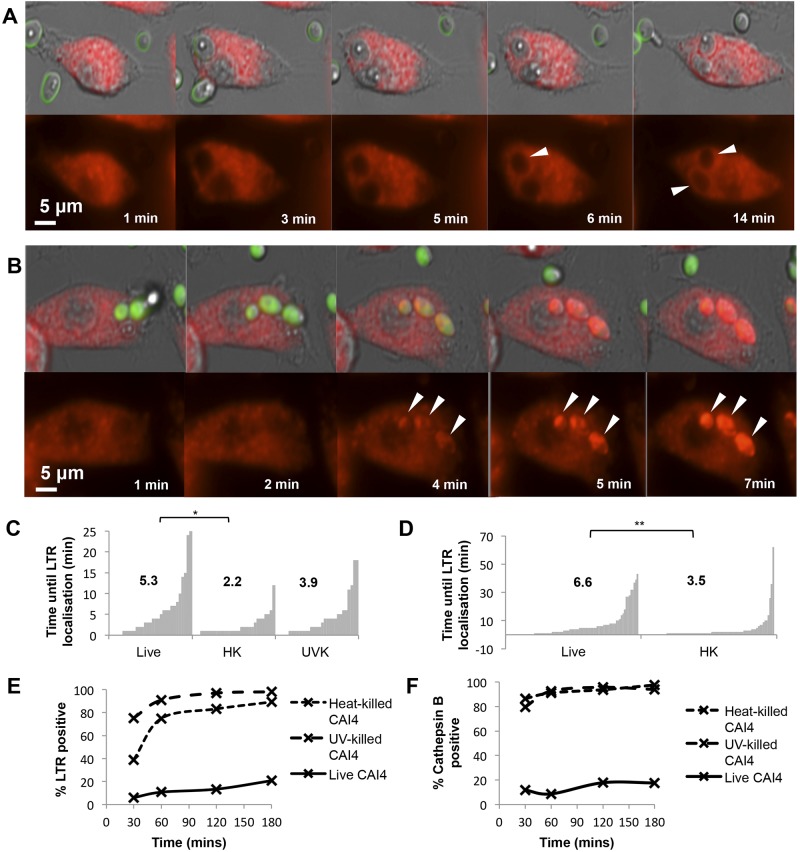
Reduced Lysotracker Red (LTR) localization to phagosomes containing live *C. albicans* cells. Observations from live-cell imaging movies (A to D) or phagoFACS (E and F) are shown. Standard phagocytosis assays were performed with murine peritoneal thioglycolate-elicited macrophages (thiomacs [A·to C]) or murine bone marrow-derived macrophages (BMDM [D]) and were imaged at 1-min intervals for 180 min. Selected movie frames are shown with LTR-stained thiomacs engulfing live (A) or heat-killed (B) CAI4 (NGY152) cells. LTR-positive phagosomes are indicated by white arrowheads, and the scale is indicated by white bars. Corresponding movies can be found in the supplemental material (Movie S1 for live cells and Movie S2 for heat-killed cells). Movies were analyzed to determine the number of minutes until LTR localized to the phagosome following the point of phagocytic engulfment (>30 phagosomes). Data are presented as plots, with clusters of bars representing the ordered series of observations for a particular condition; each bar shows the time (min) taken to localize LTR to each phagosome following particle engulfment. The mean time (min) for each condition is presented in the inset (C and D). (E and F) PhagoFACS quantification of phagosomes containing live, UV-killed (UVK), or heat-killed (HK) *C. albicans* cells liberated from J774.1 macrophages after 30, 60, 120, and 180 min. (E) LTR (acidification [early phagosome maturation]). (F) Magic Red cathepsin (activated cathepsin B [phagosome-lysosome fusion]). *, *P* ≤ 0.05; **, *P* ≤ 0.01.

We have previously shown that macrophages engulf dead *C. albicans* yeast cells at a higher rate than live cells (27). Here we address the question of whether the viability of internalized *C. albicans* impacts the rate of phagosome maturation postengulfment. To detect changes in pH in phagosomes containing *C. albicans*, we utilized the acidotropic reagent Lysotracker Red DND-99 (LTR), a weak base dye that permeates cellular membranes but is retained upon protonation, thus labeling acidic compartments. We examined the live-cell dynamics of LTR localization to thioglycolate-elicited murine macrophages (thiomacs) incubated with live, UV-killed, or heat-killed wild-type *C. albicans* strain NGY152 (CAI4 + CIp10 [here referred to as CAI4]). Video microscopy revealed that individual phagosomes containing live *C. albicans* cells took longer to localize LTR following engulfment (Fig. 1). LTR stained the phagosome peripheral to live fungal particles, suggesting LTR was excluded by the fungal cell wall (Fig. 1A; see Movie S1 in the supplemental material). Phagosomes containing dead *C. albicans* cells underwent more rapid LTR localization, which penetrated the fungal particle, suggesting an increase in fungal cell wall permeability and reduction in cell wall integrity (Fig. 1B; see Movie S2 in the supplemental material).

Phagosomes containing live *C. albicans* cells took a mean time of 5.3 min to localize LTR—significantly longer than phagosomes with heat-killed fungi, which took a mean of 2.2 min (Fig. 1C). UV-killed *C. albicans* cell-containing phagosomes took 3.9 min to localize LTR, faster than those containing live fungi (Fig. 1C). Similar data were obtained from movies of bone marrow-derived macrophages (BMDM) incubated with live and heat-killed *C. albicans* cells in which phagosomes took 6.6 min versus 3.5 min, respectively, to become LTR positive (Fig. 1D).

Phagosomes from macrophage cell line J774.1 were quantified in a cell-free phagosome cell sorter (PhagoFACS) assay by isolating phagosomes containing fluorescein isothiocyanate (FITC)-stained *C. albicans* cells and measuring LTR association following uptake periods of 30, 60, 120, and 180 min. PhagoFACS confirmed that significantly more LTR-positive phagosomes were observed following uptake of UV-killed or heat-killed *C. albicans* cells at each of the time points (Fig. 1E), even the 30-min time point, when a low proportion (6%) of phagosomes with live fungi were stained compared with those containing heat-killed or UV-killed *Candida* cells (39% and 75% of phagosomes, respectively). This difference was observed throughout the experiment, and after 180 min, only 21% of the phagosomes containing live *C. albicans* cells were positive for LTR, while almost all (89 to 98%) of those containing dead fungi were LTR positive (Fig. 1E).

As phagosome maturation proceeds, acid protein hydrolases, including cathepsins, are delivered to the phagosome by lysosomes (6). We assessed activated cathepsin B by PhagoFACS analysis, and the levels were substantially different between populations of phagosomes containing live or dead *C. albicans* cells (Fig. 1F). At the 30-min time point and throughout the experiment, approximately 80 to 97% of phagosomes containing heat-killed or UV-killed fungal cells were positive for activated cathepsin. However, only 9 to 18% of phagosomes containing live cells were positive for this marker (Fig. 1F). Taken together, these data show that, relative to dead cells, phagocytosis of viable *C. albicans* cells led to delayed acidification and delivery of activated cathepsin in the phagosome maturation program.

### *C. albicans O*-mannan affects phagosome maturation in macrophages.

**FIG 2:**
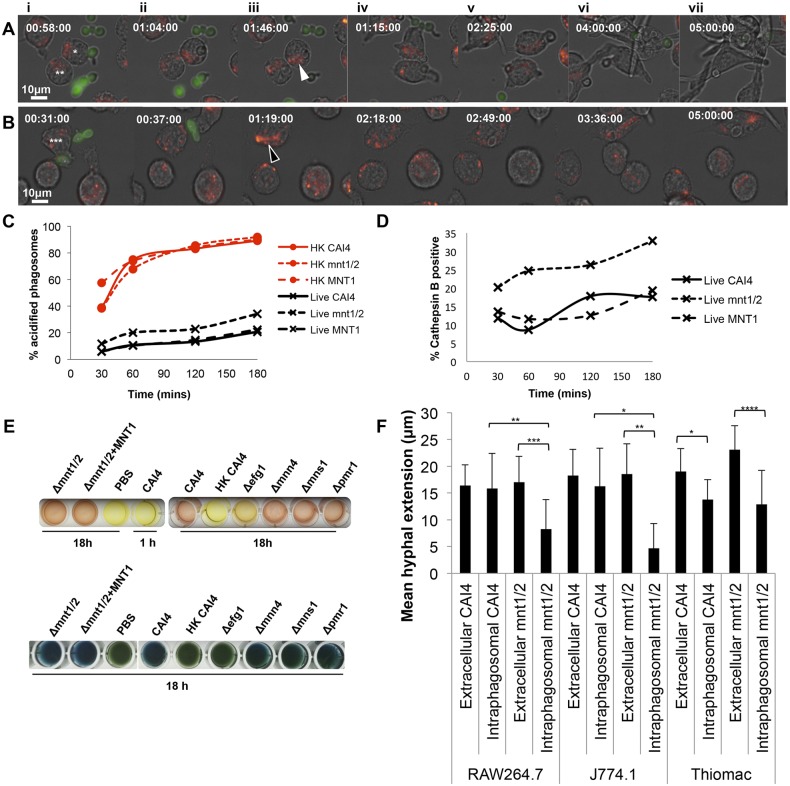
Enhanced LTR association and diminished intraphagosomal hyphal morphogenesis of the *C. albicans* Δ*mnt1*/*2 O*-glycosylation mutant. Shown are selected frames from a movie generated from images acquired at 1-min intervals for 5 h following phagocytosis of FITC-labeled cells of *C. albicans* CAI4 (NGY152) (A) and the Δ*mnt1*/2 mutant (NGY337) (B) by Lysotracker Red (LTR)-stained J774.1 macrophages. The times shown in the top left of each panel are presented as h:min:s. The scale is shown in image Ai by the white bar. In images Ai and Aii, the macrophage labeled * in Ai recognizes, binds, and phagocytoses a CAI4 yeast cell with a small germ tube. In image Aiii, faint LTR localization is observed around the hypha within the macrophage labeled * in Ai (white arrowhead). In image Aiv, the hypha in the macrophage labeled * in Ai continues to extend, meanwhile the macrophage labeled ** in Ai engulfs a yeast with a small hypha. Image Av shows LTR localization around the hyphae within both macrophages, which continue to extend. In image Avi, the macrophage labeled * in Ai is ruptured, meanwhile the macrophage labeled ** in image Ai contains hypha (>30 µm), which starts to bend within the host cell. In image Avii at 5 h, several surrounding macrophages have also been ruptured, and escaped hyphal filaments are present. In images Bi and Bii, the macrophage labeled *** in Bi recognizes, binds, and phagocytoses the Δ*mnt1*/*2* mutant cell, which forms a small germ tube. Image Biii shows intense LTR localization around the hypha (>12 µm [hollow arrowhead]). In image Biv, a second hypha emerges from the internalized yeast (~2 µm). In image Bv, the second hypha continues to grow (11 µm). Image Bvi shows intense LTR around the second hypha (16 µm) within the macrophage labeled *** in Bi. In image Bvii, there is no further growth of either hypha by 5 h. Movies for panels A and B can be found in the supplemental material (see Movie S3 and Movie S4, respectively). (C and D) PhagoFACS quantification of isolated phagosomes containing live, UV-killed, or heat-killed *C. albicans* cells from CAI4, the Δ*mnt1*/*2* mutant, and the Δ*mnt1*/*2 MNT1* mutant (NGY335) liberated from J774.1 macrophages after 30, 60, 120, and 180 min. Panel C shows LTR (acidification [early phagosome maturation]), and panel D shows Magic Red cathepsin (activated cathepsin B), indicating phagosome-lysosome fusion (late phagosome maturation). (E) Environmental alkalinization capacity of *C. albicans* strains, determined by growth in phenol red liquid (upper panels) and bromocresol green agar (middle and lower panels). (F) Movies were analyzed using the Volocity line tool to measure hyphal lengths and calculate the mean hyphal extension of extracellular and phagocytosed CAI4 (NGY152) or Δ*mnt1*/*2* (NGY337) cells over an 80-min incubation with RAW264.7, J774.1, and murine thioglycolate-elicited peritoneal macrophages (thiomacs). Data from *n* = 15 to 20 hyphae per condition were examined from multiple replicate movies. *, *P* ≤ 0.05; **, *P* ≤ 0.01; ***, *P* ≤ 0.001; ****, *P* ≤ 0.0001.

Previously, we demonstrated that a Δ*mnt1*/*2 O*-mannosylation mutant of *C. albicans* displays altered phagocytosis kinetics with enhanced uptake into macrophages but a reduced capacity for the mutant to kill the phagocyte (24, 27). We therefore hypothesized that macrophage phagosome maturation was altered following uptake of this mutant. Truncation of *O*-mannan chains in the Δ*mnt1*/*2* cell wall influences the structure of most GPI proteins in the cell wall and hence properties such as adhesion and virulence (36). Alterations in these proteins or the lack of *O*-mannan could affect engagement with phagocytic receptors and downstream aspects of phagosome maturation. We observed that the *C. albicans* Δ*mnt1*/*2* mutant (NGY337) initiated filamentation normally within the phagosome. However, LTR accumulation around the phagosome was seen to occur more intensively than for the wild type. Representative movies of LTR-stained J774.1 cells interacting with CAI4 or the Δ*mnt1*/*2* mutant are depicted by selected frames (Fig. 2A and B); the corresponding movies can be viewed in the supplemental material (Movie S3 for CAI4 and Movie S4 for the Δ*mnt1*/*2* mutant). In summary, when wild-type *C. albicans* was phagocytosed, LTR staining of the phagosome was weak and intraphagosomal hyphal extension was rapid. After 5 h, the majority of yeast cells had produced long filaments and escaped from within macrophages (Fig. 2A; see Movie S3). In contrast, phagocytosis of the Δ*mnt1*/*2* mutant, despite normal early germ tube formation, resulted in strong LTR association around hyphae, which extended less, resulting in fewer *C. albicans* cells escaping from macrophages (Fig. 2B; see Movie S4). The change in LTR dynamics was less marked than that observed between live and dead *C. albicans* cell cargoes. Phagosomes containing Δ*mnt1*/*2* cells acidified the phagosome lumen more rapidly than the wild type, taking an average of 0.79 min compared to 1.06 min. Analysis of movies with LTR-stained thiomacs phagocytosing CDH15 Δ*mnn4*/Δ*mnn4* (phosphomannan-deficient) (39) and NGY355 Δ*pmr1*/Δ*pmr1* (*O*-, *N*-, and phosphomannan-deficient) mutants (40) demonstrated that isolated loss of phosphomannan did not influence LTR localization (which took 1.11 min), but loss of *N*- and *O*-mannans led to more rapid LTR acquisition, taking on average 0.43 min (data not shown).

The PhagoFACS assay confirmed that phagosome maturation (indicated by LTR or activated cathepsin B) differed following uptake of the wild type and Δ*mnt1*/*2* mutant into J774.1 macrophages. A low proportion of phagosomes containing live control strain cells were LTR positive during the time course: 6% at the 30-min time point, increasing to 21 to 22% by 180 min (Fig. 2C). However, a greater proportion of phagosomes containing the *O*-mannan mutant strain were LTR positive throughout (initially 12% rising to 34%), and this difference was abrogated when fungi were UV killed (Fig. 2C). Intraphagosomal pH measurements determined from FITC fluorescence calibration curves constructed for each strain revealed that phagosomes containing Δ*mnt1*/2 cells were on average 1 pH unit lower than those containing wild-type cells (data not shown). Recent findings suggesting that fungal mannosylation mutants are impaired for environmental alkalinization (41) led us to assess the capacity of these strains to alkalinize their environment in response to growth on amino acids. Neither the Δ*mnt1*/*2* mutant nor other mannan mutants tested were deficient in their ability to alkalinize the media used. Overnight growth in liquid phenol red medium or solid bromocresol green medium led to alkalinization comparable to that of the wild-type strain (Fig. 2E). Between 9% and 19% of phagosomes with live control strains accumulated activated cathepsin during the course of the experiment (Fig. 2D), but significantly more phagosomes were positive for activated cathepsin after phagocytosis of Δ*mnt1*/*2* cells (Fig. 2D). Therefore, *O*-mannan contributes to the impairment of phagosome maturation following uptake of live *C. albicans* cells.

### *O*-Mannan-deficient *C. albicans* cells exhibit diminished intraphagosomal hyphal extension.

Movies of macrophages phagocytosing live Δ*mnt1*/*2 C. albicans* cells showed hyphal extension was reduced when there was strong LTR association around hyphae (Fig. 2B). The hyphal length of *C. albicans* was monitored over an 80-min period in movies showing engulfment by RAW264.7 and J774.1 cells and thiomacs using Volocity video analysis tools. CAI4 hyphae grew as efficiently inside the phagosome as extracellularly (Fig. 2F). The hyphae of the nonphagocytosed Δ*mnt1*/*2* mutant grew as efficiently as those of the wild type in proximity to macrophages; however, hyphal elongation within phagosomes was inhibited (Fig. 2F; see Fig. S1 in the supplemental material). Growth of both CAI4 and Δ*mnt1*/*2* hyphae inside thiomacs was impaired to some extent—the latter more so (Fig. 2F). Hyphal growth rates were compared at 0 to 40 min and 40 to 80 min postengulfment, revealing that the diminished intraphagosomal growth of Δ*mnt1*/*2* mutant hyphae comes from a deceleration over time and not a low rate from the outset (not shown). In summary, live-cell imaging demonstrated a reduction of intraphagosomal hyphal growth for *C. albicans* Δ*mnt1*/*2* cells, suggesting than *O*-mannan-deficient fungal cells are impaired in their ability to grow hyphae within macrophage phagosomes, which impacts their ability to escape from macrophage phagosomes.

### Intraphagosomal hyphal extension delays late phagosome maturation.

We then used markers of vesicular traffic to further probe how phagosome maturation was affected in response to phagocytosis of *C. albicans* cells. Green fluorescent protein (GFP) and red fluorescent protein (RFP) fusions of key phagosome maturation regulators, Rab5 and Rab7 GTPases, were constructed to visualize localization during live-cell imaging of phagocytosis. Newly formed phagosomes rapidly recruit Rab5 through fusion events with early endosomes bearing this specific Rab (42). Subsequently, the phagosome interacts with late endosomes, facilitated by Rab conversion, during which Rab5 and Rab7 are exchanged under the regulation of class C VPS/HOPS complex (43).

**FIG 3:**
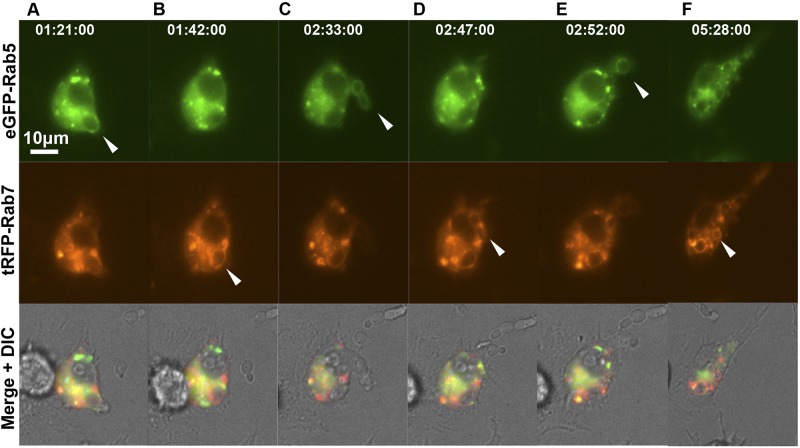
Sequential localization of Rab5 and Rab7 during live-cell imaging of phagosome maturation. Shown are RAW264.7 macrophages coexpressing eGFP-Rab5 and tRFP-Rab7. Transfected macrophages were incubated with the *C. albicans* Δ*efg1* mutant (CA79), and the temporal kinetics of Rab5 and Rab7 localization to individual phagosomes were observed at 1-min intervals. Selected frames are shown with times indicated in the upper panels (h:min:s). White arrowheads indicate the localization of Rab5 to newly engulfed *C. albicans* cells (upper row), with sequential loss of the Rab5 signal and acquisition of the Rab7 signal (middle panels), which occurs throughout the movie, during which several fungal particles are sequentially engulfed. The corresponding movie is available in the supplemental material (see Movie S5). The scale is shown by a white bar.

**FIG 4:**
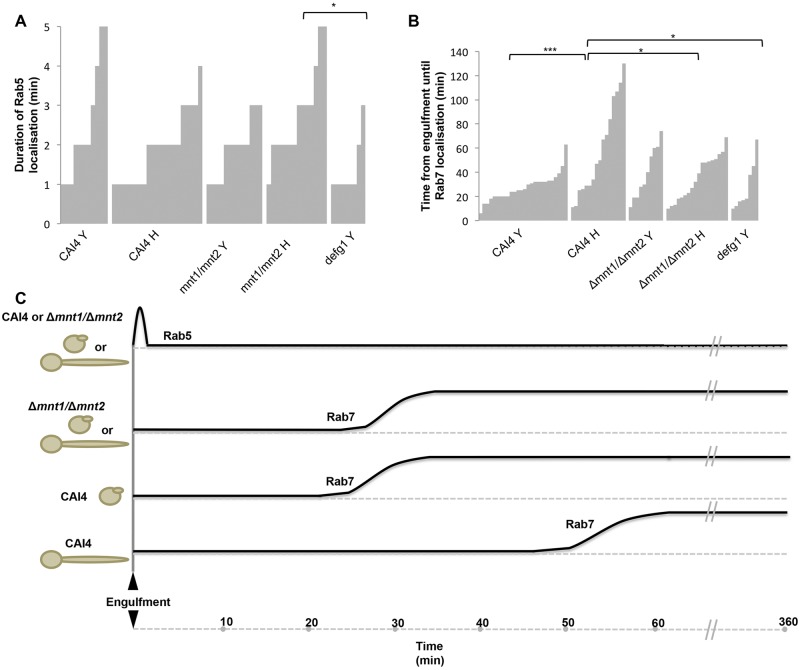
Kinetics of Rab5 and Rab7 localization to phagosomes containing *C. albicans*. The dynamics of phagosome maturation markers Rab5 and Rab7 were monitored for >70 events of *C. albicans* phagocytosis during 1-min-interval live-cell imaging of RAW264.7 cells transfected with eGFP-Rab5 and tRFP-Rab7 phagocytosing live cells of CAI4 (NGY152), the Δ*mnt1*/*2* mutant (NGY337), and the Δ*efg1* mutant (CA79). Data are presented as plots with clusters of bars representing the ordered series of observations for a particular condition; each bar shows the time (min) for an individual phagosome. Panel A shows the total duration of Rab5 retention on phagosomes containing *C. albicans* cells. Panel B shows the time taken between engulfment of the particle and Rab7 recruitment to phagosomes. *, *P* ≤ 0.05; ***, *P* ≤ 0.001. (C) Schematic diagram summarizing differential localization kinetic profiles for Rab5 and Rab7 on phagosomes containing *C. albicans* cells.

Plasmids to express enhanced GFP (eGFP)-Rab5 and turbo RFP (tRFP)-Rab7 were transfected into RAW264.7 macrophages individually or in combination. Cotransfected Rab reporter macrophages exhibited sequential localization of Rab5, followed by Rab7 to the phagosome, which is seen most clearly during phagocytosis of strain CA79, a nonfilamentous Δ*efg1*/Δ*efg1* mutant (44) (Fig. 3; see Movie S5 in the supplemental material). This has been previously described for other pathogens, although Rab5 and Rab7 dynamics have not been studied in live phagocytes processing fungi. Following the uptake of *C. albicans* by macrophages, Rab5 localized early to the phagosome (Fig. 3A, upper panel, arrowhead). Rab5 localization was transient, replaced with Rab7 (Fig. 3B, middle panel, arrowhead), which remained on the phagosome for the duration of the 6-h movie (Fig. 3C to F, middle panels). In this specific example, the same macrophage phagocytosed further *C. albicans* Δ*efg1*/Δ*efg1* yeast cells, and these phagosomes exhibited comparable sequential Rab5 and Rab7 localization kinetics (Fig. 3C and E, upper panels, and D and F, middle panels, arrowheads). Rab7 fluorescence became increasingly intense with time (Fig. 3F, middle panel). Rab5 localized to phagosomes almost immediately following engulfment of various strains, and all phagosomes had recruited Rab5 within the first 3 min postengulfment irrespective of contents (data not shown). Rab5 localization was transient, remaining on phagosomes for 1 to 5 min, with no discernible differences among the different strains or morphotypes phagocytosed (Fig. 4A). The average duration for Rab5 localization was approximately 2 to 3 min. Therefore, Rab5 localization occurs very early after phagocytosis of *C. albicans*, and the kinetics of localization of this early stage marker for phagosome maturation were not influenced by hyphae or *O*-mannan. Rab7 localization to the phagosome was then analyzed as a marker for late-stage phagosome maturation. Uptake of live hypha-competent CAI4 yeast cells and yeast-locked Δ*efg1*/Δ*efg1* cells exhibited comparable Rab7 kinetics. Rab7 was recruited as soon as 10 min postengulfment and as late as around 65 min (Fig. 4B), with averages of 27 ± 11.3 min and 28 ± 20.2 min, respectively. However, when CAI4 had already formed hyphae at the time of phagocytosis, the recruitment of Rab7 was substantially delayed. In this case, Rab7 was recruited as early as 10 min, but this could be delayed until 130 min (Fig. 4B), with an overall average of 59 ± 38.8 min. Interestingly, the Rab7 kinetics surrounding phagosomes containing the Δ*mnt1*/*2* mutant were slightly delayed, compared with those in wild-type yeast, taking 39 ± 21.4 min (Fig. 4B). However, engulfed Δ*mnt1*/*2* mutant cells that had already formed hyphae failed to delay Rab7 recruitment (Fig. 4B), taking an average of 36 ± 18.2 min for Rab7 recruitment, compared to 59 min in the wild type. Combined these data demonstrate that hyphal extension disrupts phagosome maturation, in particular the trafficking regulator Rab7, as summarized in the schematic diagram in Fig. 4C.

### Altered macrophage actin dynamics around phagosomes are affected by fungal *O*-mannan.

**FIG 5:**
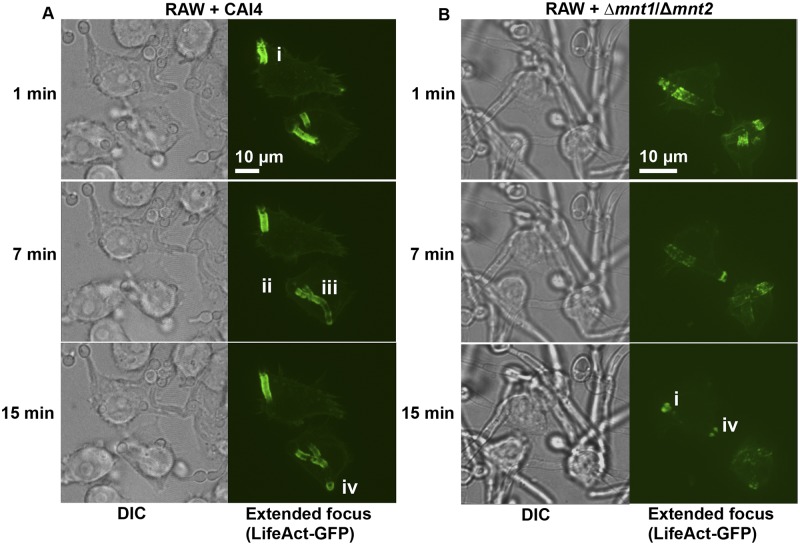
Diminished actin polymerization around phagosomes containing the *C. albicans* Δ*mnt1*/*2* mutant cells during live 4D imaging. RAW264.7 cells transfected to express LifeAct (an actin binding peptide-GFP fusion) were incubated with live *C. albicans* cells of strain CAI4 (NGY152) (A) or the Δ*mnt1*/*2* mutant (NGY337) (B) for 2 h prior to 4D movie acquisition with 55 z slices imaged at 50-s intervals for 20 to 40 min. Selected time point frames from movies (1 min, 7 min, and 15 min) are shown as composites of z-stacks (extended focus) for GFP images (right) or a representative single z-stack from DIC (left). Actin polymerization is shown at macrophage entry points (i), faintly around internal yeast phagosomes (ii), lining internal hyphal phagosomes (iii), and at macrophage exit/distension points (iv). The scale is shown by white bars. Corresponding movies can be viewed in the supplemental material (see Movie S6 and Movie S7).

**FIG 6:**
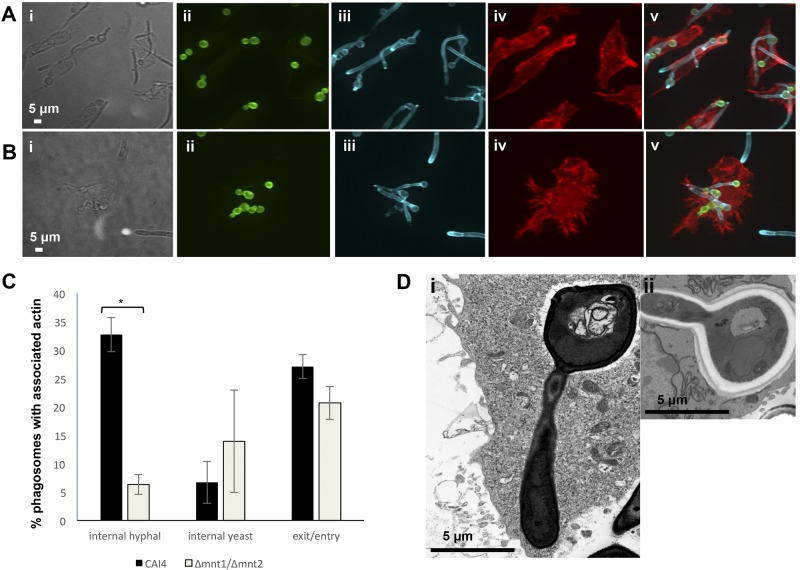
Diminished actin polymerization around phagosomes containing hyphae of the *C. albicans* Δ*mnt1*/*2* mutant imaged in 3D. RAW264.7 cells were combined with live *C. albicans* cells from strain CAI4 (NGY152) (A) or the Δ*mnt1*/*2* mutant (NGY337) (B) in standard phagocytosis assays for 2 h prior to fixing of cells, staining, and imaging. The scale is shown by white bars. Representative DIC (i) and composite *z*-stack (ii to v) images are shown: extended focus (images ii to v) is shown following phagocytosis of both strains. Cells were stained as follows: prephagocytosis FITC staining of *C. albicans* cells (ii), postfix CFW staining of fungal cell wall chitin (iii), postfix macrophage phalloidin staining of actin (iv), and merge (v). (C) Quantification of polymerized actin associated with phagosomes containing *C. albicans* determined from analysis of >90 phagosomes (% association with internalized hyphal or yeast parts of *C. albicans* phagosomes or with entry or exit points of the macrophage). *, *P* ≤ 0.05. (D) Differential phagosomal membrane apposition surrounding yeast and hyphal parts of *C. albicans* wild-type strain CAI4 within J774.1 (i) and RAW264.7 (ii) cells visualized by TEM.

We next examined actin polymerization dynamics around phagosomes containing live wild-type and *O*-mannan-deficient *C. albicans* cells. RAW264.7 macrophages were transfected with LifeAct (a plasmid encoding an actin binding peptide-GFP fusion) prior to phagocytosis of *C. albicans*, following which live-cell imaging captured actin dynamics in 4 dimensions, with z-stacks generated at 50-s intervals. Four patterns of actin polymerization were observed around phagosomes containing CAI4 hyphae. First, intense cuffs of actin were evident at entry points at which macrophages attempted to phagocytose hyphae (Fig. 5A, i, top panel; see Movie S6, upper macrophage, in the supplemental material). Second, actin flashes were observed, often with a punctate pattern, around the yeast part of internalized CAI4 (Fig. 5A, middle panel, ii). Third, internalized hyphae were often surrounded by tubes of polymerized actin around all or most of the hyphae, and these exhibited dynamic alterations with time (Fig. 5A, middle panel, iii). Finally, regions surrounding hyphae and hyphal tips that caused points of macrophage outer membrane distension exhibited intense actin polymerization (Fig. 5A, bottom panel, iv). Actin polymerization on phagosomes containing Δ*mnt1*/*2* hyphae displayed a rapidly changing pattern (Fig. 5B; see Movie S7 in the supplemental material). Points of hyphal entry were again more intense than the actin around internalized portions of hyphae (Fig. 5B, bottom panel, i and iv). In contrast to phagosomes containing wild-type hyphae, actin polymerization around phagosomes containing the Δ*mnt1*/*2* mutant was less intense, and dynamic actin lining sections of the hypha was more punctate (Fig. 5B, upper panel). Imaging of the yeast-locked Δ*efg1*/Δ*efg1* strain revealed that although actin polymerized at the site of engulfment, internal yeast phagosomes were not associated with polymerized actin (see Movie S8 in the supplemental material). Phagocytosis assays were performed to quantify differences between actin polymerization around CAI4- and Δ*mnt1*/*2* cell-containing phagosomes. After 2 h of incubation, cells were fixed, permeabilized, and then incubated with calcofluor white (CFW) to stain the fungal cell wall (chitin) and phalloidin to stain actin. Three-dimensional sections were imaged, and composite images (generated using extended focus) were scored for the presence of polymerized actin associated with entry or distension/exit sites at the macrophage membrane, surrounding internalized yeast cells, and surrounding internalized hyphae. Representative three-dimensional (3D) images are shown in Fig. 6A and B. The percentage of phagosomes with polymerized actin lining internalized hyphae was drastically reduced following uptake of Δ*mnt1*/*2* cells: 6% compared to 33% for the wild type (Fig. 6C). Internalized yeast cells were less frequently associated with actin encapsulation, and there was no significant difference observed between phagosomes containing wild-type or mutant cells. Finally, actin was detected around entry and distension/exit points in equal proportions for phagosomes containing either strain of *C. albicans* (Fig. 6C). To further examine the phagosome encasing yeast cells and hyphae, we used transmission electron microscopy (TEM) to visualize J774.1 or RAW264.7 macrophages that had phagocytosed wild-type *C. albicans* cells. Differences were observed in the association of the phagosomal membrane around yeast and hyphal portions of germ tubes. The phagosome membrane was tightly apposed to hyphal but not yeast cell walls (Fig. 6D). These observations suggest a model in which macrophages respond to rapidly extending hyphae within phagosomes by increasing the association of polymerized actin around the internalized particle, perhaps in an attempt to contain the expanding phagosome and avoid lysis. Importantly, the cytological data also reflect the profound differences observed in hyphal growth rates between the wild type and the *O*-mannan mutant strain.

### Reduced macrophage killing capacity of the *C. albicans O*-mannosylation mutant.

**FIG 7:**
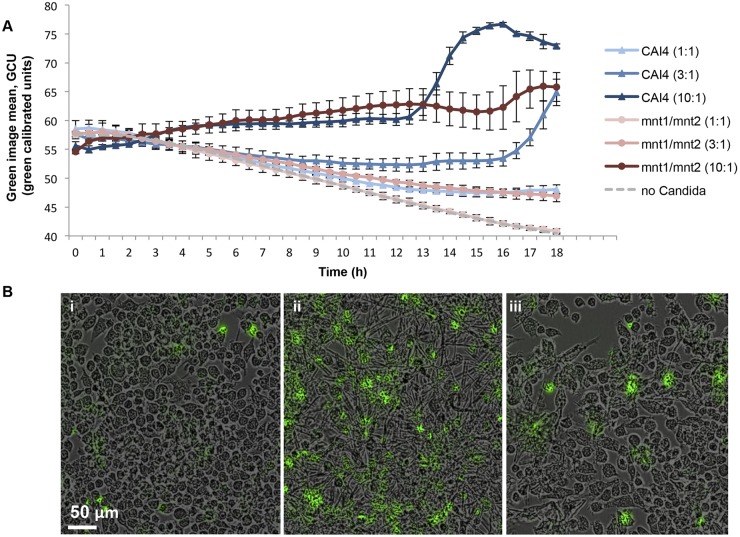
The *C. albicans* Δ*mnt1/2* mutant has reduced capacity for macrophage lysis. Phagocytosis assays were performed by combining cells from *C. albicans* strain CAI4 (NGY152) and the Δ*mnt1*/*2* mutant (NGY337) with J774.1 macrophages at multiplicities of infection (MOI) of 1:1, 3:1, and 10:1 (ratio of fungal cells to macrophages) with culture medium containing YOYO-1 dye. Images were captured at 30-min intervals during 18 h of incubation, and fluorescence generated from the binding of YOYO-1 to extracellular DNA released from lysed macrophages was detected. (A) Average image mean for YOYO-1 (green calibrated units) of *n* = 2 experiments over 18 h. (B) Representative merged DIC and YOYO-1 images taken at the end of 18 h of incubation of phagocytosis assays without *C. albicans* (i), with CAI4 at an MOI of 3:1 (ii), or with the Δ*mnt1*/*2* mutant at an MOI of 3:1 (iii). The scale is shown by a white bar.

The diminished killing capacity of the Δ*mnt1*/*2* mutant phagocytosed by J774.1 was demonstrated previously by measuring macrophage lysis at set time points (24). We confirmed this by live-cell imaging phagocytosis assays combining RAW264.7 macrophages with different multiplicities of infection (MOIs) of the wild type or Δ*mnt1*/*2* mutant in the presence of the high-affinity nucleic acid stain YOYO-1 dye. This indicator emits low fluorescence in culture medium until it is intercalated with extracellular DNA released from lysed macrophages, upon which the fluorescence intensity is increased >1,000-fold (45). The mean fluorescence intensity of each image was determined at 30-min intervals over 18 h to determine the kinetics of the killing capacities of the two *C. albicans* strains. During the time course, uninfected macrophages exhibited a net decline in signal, representing dissipating fluorescence from extracellular DNA of background dead macrophages (Fig. 7A). Increased macrophage killing as measured by YOYO-1 fluorescence was evident after 3 h of imaging and became increasingly apparent at later time points. *C. albicans* cells deficient in *O*-mannan were less able to kill macrophages; at the lowest MOI of 1:1, the Δ*mnt1*/*2* mutant strain did not generate detectable lysis beyond that of uninfected macrophages (Fig. 7A). A 3:1 MOI of Δ*mnt1*/*2* cells yielded YOYO-1 fluorescence comparable to that of the CAI4 strain at a 3-fold-lower MOI (1:1) (Fig. 7A). YOYO-1 fluorescence at a high MOI of 10:1 was comparable to those for the wild type and Δ*mnt1*/*2* mutant, but after 12 h, the reduced killing capacity of the Δ*mnt1*/*2* mutant became apparent (Fig. 7A). Representative images from movies at 18 h are shown in Fig. 7B for an MOI of 3:1. The killing capacity of *C. albicans* strains was then deduced by observing thiomacs infected with *C. albicans* at an MOI of 0:1 during live-cell imaging over 6 h. By the end of this time period, the percentage of macrophages lysed following uptake of one or more live fungal cells was markedly less for the Δ*mnt1*/*2* strain, which lysed 29% of macrophages (18 lysed out of 62 phagocytic macrophages) compared to the wild type, which lysed 82% of macrophages (32 lysed out of 39 phagocytic macrophages [data not shown]).

### Unmasking of β-glucan in the Δ*mnt1*/*2* cell wall enhances recognition by Dectin-1.

**FIG 8:**
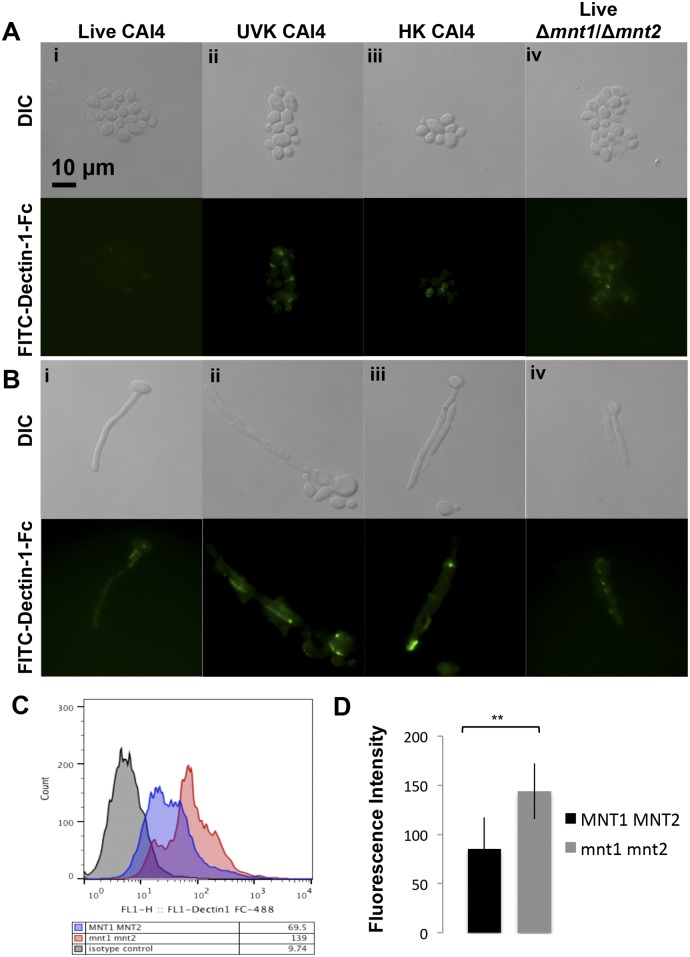
Increased β-glucan exposure on the surface of killed *C. albicans* cells and live yeast cells from the Δ*mnt1*/*2 O*-mannan mutant. The Dectin-1-Fc reporter was incubated with yeast (A) or hyphae (B) of live CAI4 (NGY152) cells (i), UV-killed (UVK) CAI4 cells (ii), heat-killed (HK) CAI4 cells (iii), or live Δ*mnt1*/*2* mutant (NGY337) cells (iv), to indicate exposure of β-glucan on the cell surface. Shown are representative images of DIC (upper panels) and Dectin-1-Fc (lower panels). The scale is indicated by the bar. (C) FACS determination of β-glucan exposure on the cell surface of wild-type SC5314 or Δ*mnt1*/*2* mutant yeast cells detected by Fc-Dectin-1. A representative histogram shows mean fluorescence intensities (black, isotype control; blue, SC5314; red, Δ*mnt1*/*2* mutant). (D) Combined data from triplicate experiments comparing SC5314 with Δ*mnt1*/*2* cells. **, *P* ≤ 0.01.

Our data show that intraphagosomal hypha formation of an *O*-mannan-deficient mutant was impaired and that phagosome maturation was retarded, which was reflected in modification of the macrophage actin dynamics and ultimately a reduced capacity to kill and escape from host cells. To further define the role of *O*-mannan in phagosome maturation, we examined whether the Δ*mnt1*/*2* mutant cell wall permits differential engagement of fungal PAMPs by host phagocytic receptors which could impact the rate of phagosome maturation. The PRR Dectin-1 recognizes the fungal cell wall component β-1,3-glucan as a PAMP (31) and was recently found to drive phagosome maturation of synthetic fungal particles comprising latex beads coated with β-1,3-glucan (46). Therefore, we used a Dectin-1-Fc reporter (ligand binding domain of Dectin-1 fused to Fc) to assess β-glucan exposure in the cell wall of different strains or preparations of *C. albicans*. Live wild-type yeast cells had very little exposed β-glucan, with staining confined to the bud scars of yeast (Fig. 8A, i) and the yeast-hypha junction of filaments (Fig. 8B, i). Exposure was far greater on the cell wall of the Δ*mnt1*/*2* mutant and was more uniform over the cell surface of yeast (Fig. 8A, iv), although hyphal Δ*mnt1*/*2* cells did not stain with Dectin-1 reporter more than the wild-type hyphae (Fig. 8B, iv). UV- and heat-killed yeast and hyphal forms of CAI4 all exposed more β-glucan (Fig. 8A, ii and iii, and 8B, ii and iii), a likely effect of opening up the cell wall, as concanavalin A (ConA) staining for mannan was enhanced in killed cells, particularly those heat killed (see Fig. S2 in the supplemental material). The difference in β-glucan exposure between wild-type and Δ*mnt1*/*2* mutant yeast cell walls was then quantified by FACS. A representative histogram is shown in Fig. 8C. Significantly greater Dectin-1 reporter bound to the Δ*mnt1*/*2* mutant than to the wild type (Fig. 8D), confirming observations made by microscopy.

### Delayed phagosome maturation in Dectin-1-deficient cells.

**FIG 9:**
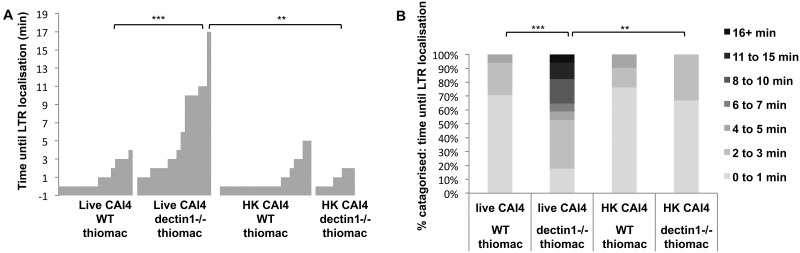
Phagosome acidification is delayed in macrophages lacking Dectin-1. Thioglycolate elicited macrophages (thiomacs) from wild-type or Dectin-1 knockout mice were prepared for standard phagocytosis assays with CAI4 (NGY152) prior to live-cell imaging. Movies were analyzed to determine the number of minutes until LTR localized to the phagosome (for >30 phagosomes). In panel A, data are presented as plots, with clusters of bars representing the ordered series of observations for a particular condition; each bar shows the time (min) taken to localize LTR to each phagosome following particle engulfment. In panel B, data are presented as a categorized chart. **, *P* ≤ 0.01; ***, *P* ≤ 0.001.

Next we tested whether loss of *C. albicans* engagement with Dectin-1 disrupted phagosome maturation. Murine thiomacs from wild-type or Dectin-1^−/−^ mice were combined with *C. albicans* cells for live-cell imaging using LTR as a readout of phagosome acidification. Following engulfment of CAI4 cells, phagosomes were significantly delayed for LTR accumulation in the Dectin-1^−/−^ cells compared to the wild type, when live fungi were phagocytosed, although this difference was lost when dead fungi were engulfed, and equally rapid LTR accumulation occurred (Fig. 9). In summary, the Δ*mnt1*/*2* mutant unmasks β-glucan in the cell wall, which is more efficiently recognized by Dectin-1. Dectin-1 recognition is required for phagosome acidification; therefore, *O*-mannan masking of β-glucan represents a mechanism by which *C. albicans* avoids more rapid phagosome maturation, allowing hyphal extension and escape from the phagocyte.

## DISCUSSION

Host immune defense against *C. albicans* relies upon the ability of macrophages to identify, phagocytose, and inactivate fungal cells, which are encountered in a variety of niche-dependent morphological forms, including yeast cells and hyphae. In the present study, we analyzed *C. albicans* factors that affect postengulfment processes. We show that viable but not dead *C. albicans* cells profoundly delay phagosome maturation, which is in part dependent upon cell wall composition and morphogenesis. Loss of cell wall *O*-mannan was associated with enhanced acquisition of phagosome maturation markers, impaired hyphal growth within macrophage phagosomes, profound changes in macrophage actin dynamics, and ultimately a reduced ability of fungal cells to escape from macrophages. Loss of cell wall *O*-mannan exposed β-glucan at the fungal cell surface, facilitating recognition by Dectin-1, which was associated with enhanced phagosome maturation.

Previously, we described component steps of phagocytosis, including migration of macrophages toward the *C. albicans* target, recognition, and engulfment, and assessed the relative contributions of viability, cell wall composition, and morphogenetic status to these processes (27). To explore downstream processes beyond engulfment of *C. albicans*, we combined live-cell imaging with a variety of cytological markers to assess phagosome maturation.

Rab5 GTPase is a key regulator present on early endosomes, which fuse with nascent phagosomes, marking the onset of early phagosome maturation (42). Rab5 is essential for phagosome maturation, during which it is replaced with Rab7, a key driver of late phagosome maturation (43). Manipulation of Rab5 and Rab7 is an immune evasion mechanism employed by several important pathogen species. For example, phagosomes containing *M. tuberculosis* cells exhibit Rab5 retention and Rab7 exclusion, thereby avoiding fusion with lysosomes (47). We describe here, for the first time, the kinetics of host Rab5 and Rab7 in the phagocytosis of *C. albicans*. Live-cell imaging of eGFP-Rab5-transfected macrophages revealed localization of this GTPase to phagosomes containing *C. albicans* cells almost immediately upon phagocytosis. The majority of phagosomes were Rab5 positive within the first minute postengulfment. Rab5 was retained transiently for approximately 2 min, but never more than 5 min. Our data show that Rab5 kinetics following phagocytosis of *C. albicans* are independent of fungal morphology or *O*-mannan status. We also rule out Rab5 retention as a mechanism utilized by *C. albicans* to subvert phagosome maturation.

Phagosome interactions with endocytic vesicles facilitate v-ATPase recruitment to the phagosome membrane, generating a lumenal pH drop from 6.5 to 5.0 within minutes (48), which can be visualized by live-cell imaging of macrophages stained with acidotrophic LTR. Our data are in agreement with those from other studies where heat-killed yeast cells are associated with maturation markers at early time points (19, 20). Following engulfment, phagosomes containing live *C. albicans* cells were delayed for LTR labeling compared to those containing dead fungi. PhagoFACS experiments expanded upon the LTR data from live-cell imaging, showing that more phagosomes were LTR positive at all time points if dead fungi were engulfed. Similar results were obtained for activated cathepsin B, which transits from lysosomes and becomes active at lower pH. The advantage of analyzing movies is that dynamic changes of individual phagosomes can be determined rather than population-based averages from a single time point, where phagocytosis events can occur asynchronously. Furthermore, differences in LTR and cathepsin B staining are exaggerated in phagoFACS experiments due to cell wall permeabilization of dead fungi, which enhances staining. Nonetheless, population-based methods such as phagoFACS complement live-cell analyses.

Compared to wild-type *C. albicans* cells, Δ*mnt1*/*2* mutant cells are engulfed by J774.1 macrophages more slowly, but in greater numbers; however, this strain is less able to kill phagocytes (24, 27). Indeed, acidification of phagosomes containing Δ*mnt1*/*2* cells occurred earlier in both thiomacs and J774.1 cell line macrophages. PhagoFACS confirmed enhanced LTR and activated cathepsin B labeling of phagosomes containing the *O*-mannan mutant. Intraphagosomal determination of pH calculated from FITC fluorescence revealed Δ*mnt1*/*2* cell-containing phagosomes are 1 pH unit below those containing the wild-type *C. albicans* cells, confirming not only a temporal delay in the early minutes of phagosome acidification but a quantitative change that can be detected after several hours. Recently an amino acid permease of *C. albicans*, Stp2, was found to be required for intraphagosomal neutralization and subsequent hyphal growth in *C. albicans* (22). Mannosylation genes *MNN10* and *MNN11* of the related species *Candida glabrata* were identified as having a role in environmental alkalinization during growth on amino acids (40), nutrient conditions that are thought to mimic the phagosomal environment. However, Δ*mnt1*/*2* cells were proficient in alkalinization, suggesting that failure to neutralize the compartment is not the cause of enhanced phagosome acidification. Intraphagosomal hyphal growth of the Δ*mnt1*/*2* mutant, but not CAI4, decelerated from an initial normal rate following engulfment. Interestingly, thiomacs were able to somewhat slow the hyphal growth of wild-type *C. albicans*, but to a lesser degree than for the Δ*mnt1*/*2* mutant, despite the differences in LTR kinetics observed in the same cells, highlighting subtleties in the relationships between acidification and hyphal growth.

Enhanced acidification of Δ*mnt1*/*2* cell-containing phagosomes did not advance Rab7 localization to phagosomes, a marker that indicates fusion of phagosomes with late endosomes. However, macrophages that ingested live wild-type *C. albicans* cells were markedly delayed in Rab7 localization to phagosomes if the fungal cell had already initiated hyphal morphogenesis prior to engulfment. This hypha-mediated delay of phagosome maturation is in agreement with previous work where late-stage markers were detected less frequently in macrophages containing hyphal *C. albicans* at various time points (19). We speculate that the rapid physical expansion of such phagosomes interferes with the complex and transient trafficking interactions that culminate in Rab7 acquisition on the phagosome. Macrophages phagocytosing *M. tuberculosis* are manipulated by the pathogen to retain phagosomal Rab14, promoting early endosome-phagosome and phagosome-phagosome fusion events, which generates additional membrane to support the burgeoning mycobacterium-containing vacuole and accommodate multiplying bacteria (49). A rapidly growing *C. albicans* hypha may induce alterations within the host cell to accommodate fungal growth, thereby preventing phagosomal rupture and release of a cytotoxic cache. Our TEM observations revealed a differentially apposed phagosomal membrane—tight around hyphae but loose around yeast cells—highlighting requirements upon phagosomes regions to contain expanding hyphae.

We further explored the host response to phagocytosed hyphae by examining actin dynamics using 3D and 4D microscopy. Previous work has suggested that the dynamic localization of polymerized actin surrounding *C. albicans* hypha-containing phagosomes may assist the fungus to escape (50). Other studies describe “actin flashing” in macrophages phagocytosing beads as a mechanism to block lysosome docking, thereby delaying phagosome maturation (51). In this work, we observed intense F-actin staining at sites of hyphal internalization, representing the actin “cuff” regions described elsewhere (35). We observed from 4D movies peristaltic wave-like dynamics of cuffs around hyphae at points of internalization, suggesting a mechanism to draw filaments into the macrophage. Such a mechanism may promote optimum actin function, fitting with our previous finding: end-on hyphae are internalized more rapidly than alternatively encountered geometries (27). Actin was dynamically remodeled around the growing *C. albicans* cell-containing phagosome, particularly around internalized hyphae, but less so surrounding the yeast part, where a more punctate or cage-like pattern was seen. We speculate that actin polymerization occurs in response to rapidly growing fungal cells, since dead hyphae (not shown) and a live yeast-locked Δ*efg1*/Δ*efg1* mutant did not elicit this pattern of F-actin. In line with this hypothesis, phagosomes containing the Δ*mnt1*/*2* mutant were surrounded by actin that appeared far less intense and exhibited punctate decoration around hyphal phagosomes, presumably in response to decelerated intraphagosomal hyphal growth. Whether actin polymerization around growing *C. albicans* hyphae is a mechanism that confers a survival advantage to the pathogen or the host remains to be determined.

The fungal pathogen *Cryptococcus neoformans* undergoes nonlytic expulsion from phagocytes, which may facilitate immune evasion and dissemination in the host (52, 53). Actin flashes around phagosomes containing cryptococci have been described as a host-driven mechanism to prevent escape of these fungi from host cells (54). We described previously nonlytic expulsion of *C. albicans* from macrophages *in vitro* (55), although the role of actin during such events remains to be defined.

We have described that following internalization of Δ*mnt1*/*2* cells, the phagosome is differentially processed: acidification is more rapid, activated cathepsin accumulates more readily, and hyphal extension is retarded. We have confirmed that, consequently, fewer phagocytes are lysed, as described using a different methodology earlier (24). These data support a mechanism of hypha-mediated physical rupture of macrophages, although hyphal induction of pyroptosis has recently been described as a further mechanism by which *C. albicans* kills host cells (56, 57), adding modulation of death pathways to a repertoire of mechanisms pathogens utilize to their advantage (58). In addition to previous work showing an altered engulfment rate of the Δ*mnt1*/*2* mutant (27), the present work indicates altered recognition by phagocyte pattern recognition receptors (PRR) under opsonin-free conditions. Previously, *N*-mannan mutants of *C. albicans* have been shown by TEM to display truncated cell wall fibrils and expose β-glucan in the cell wall (29). Recognition of β-glucan by the PRR Dectin-1 mediates phagocytosis of fungal particles and initiation of proinflammatory responses, representing an important interaction for immunity to fungal infection (59, 60). Here we show that Δ*mnt1*/*2* yeast cells expose greater β-glucan at their surface and that soluble Dectin-1 is able to access and recognize this polymer. Recently Dectin-1-dependent activation of spleen tyrosine kinase (Syk) was shown to drive the phagosome maturation of β-1,3-glucan-coated beads (46), thereby defining Dectin-1 as a regulator of phagosome maturation. The unmasking of β-glucan on the fungal cell surface of the *O*-mannan mutant may permit enhanced binding of Dectin-1 during phagocytosis, driving more rapid phagosome maturation. Indeed, live *C. albicans* cells engulfed by thiomacs from Dectin-1^−/−^ knockout mice were substantially delayed for phagosomal acidification. Only a slight delay in acidification was observed in Dectin-1^−/−^ thiomacs phagocytosing killed yeast cells with exposed cell surface β-glucan. One explanation for this is that there are separate mechanisms underpinning subversion of phagosome maturation by *C. albicans*. First, *O*-mannan contributes to the masking of underlying β-glucan in the yeast cell wall, thereby blocking recognition by Dectin-1 with downstream consequences on phagosome maturation. Second, the capacity for hyphal extension (promoted by alkalinization [22]) delays phagosome maturation and has a significant impact upon actin dynamics and killing of host cells. Both aspects are relevant *in vivo* during *C. albicans* infection. Increased β-glucan exposure occurs on yeast cells and hyphae during infection of organs in mice and on hyphae only upon exposure to the antifungal drug caspofungin (34). Additionally, host niche-specific carbon sources influence cell wall structure (61), generating phenotypic variability, which will translate into differential processing postengulfment. This work refines our understanding of the complex interplay at the host-pathogen interface and reinforces the importance of hyphal growth and cell wall composition in *C. albicans* pathogenicity by demonstrating how these features affect phagosome maturation and the efficiency of the innate immune response to fungi.

## MATERIALS AND METHODS

### Ethics statement.

This work was approved by the Animals Scientific Procedures Division, Home Office, United Kingdom Government under the personal license of Lars Erwig (PIL60/6194) and Project Licence PPL60/4007, in line with European Union Directive 2010/63/EU on the Protection of Animals Used for Scientific Purposes.

### Preparation of murine primary cells.

Experiments were approved by the College of Life Sciences and Medicine Ethics Review Board, University of Aberdeen with adherence to local and institutional policy requirements. C57BL/6 wild-type and Dectin1^−/−^ knockout mice were bred and housed in the University of Aberdeen animal facility. Female mice aged 8 to 10 weeks were used to generate primary cells. Thioglycolate-elicited peritoneal macrophages (thiomacs) were harvested from sacrificed mice 4 days after a 1-ml injection of Brewer’s thioglycolate broth (Becton Dickinson, Franklin Lakes, NJ) to the peritoneal cavity. Thiomacs were lavaged in 5 ml of 5 mM EDTA in phosphate-buffered saline (PBS), kept on ice, and then washed 3 times with RPMI 1640 (Sigma, Dorset, United Kingdom) supplemented by 10% (vol/vol) heat-inactivated fetal calf serum (FCS) (Biosera, Ringmer, United Kingdom), 200 U ml^−1^ penicillin-streptomycin (Invitrogen Ltd., Paisley, United Kingdom), 10 mM HEPES (Invitrogen) and 2 mM l-glutamine (Invitrogen). Bone marrow-derived macrophages (BMDM) were prepared by flushing femurs and tibia with supplemented RPMI and then washing 3 times with the same medium. Following cell counts, 1.5 × 10^5^ thiomacs or bone marrow cells in 300 µl supplemented RPMI medium were seeded onto 8-well imaging slides (ibidi, Munich, Germany) and cultured overnight at 37°C with 5% CO_2_, after which nonadherent cells were removed by replacing medium. Thiomacs were used for experiments 1 day postharvest, and bone marrow cells were cultured for 5 days to promote differentiation into BMDM.

### Preparation of murine macrophage cell lines.

Cells of the J774.1 and RAW264.7 murine macrophage lines (European Collection of Cell Culture) were passaged and cultured at 37°C and 5% CO_2_ in Dulbecco’s modified Eagle’s medium (DMEM) (Lonza, Braine-l’Alleud, Belgium), supplemented as described above. Cells were seeded at 1.2 × 10^5^ cells/well in an 8-well slide or at 1 × 10^6^ cells/dish in a 35-mm dish (ibidi) and cultured overnight at 37°C with 5% CO_2_.

### Preparation of *C. albicans* cells.

Strains were thawed from −80°C glycerol stocks and grown at 30°C on solid yeast extract-peptone-dextrose (YPD) prepared from 1% (wt/vol) yeast extract (Oxoid, Cambridge, United Kingdom), 2% (wt/vol) peptone (Oxoid), 2% glucose (Fisher Scientific, Leicestershire, United Kingdom), and 1.5% Technical agar (Oxoid), or synthetic complete medium lacking uracil (SC −Ura) prepared from 0.69% (wt/vol) yeast nitrogen base-amino acids (Formedium, Norfolk, United Kingdom), 1.5% (wt/vol) technical agar (Oxoid), 0.1% (vol/vol) of 1 M NaOH solution (BDH Chemicals, VWR International, Leicestershire, United Kingdom), 0.01% (vol/vol) adenine hemisulfate (Sigma), 4% (wt/vol) glucose (Fisher Scientific), and 0.4% (wt/vol) SC −Ura dropout amino acid mix (Formedium). A colony of *C. albicans* was inoculated into liquid YPD or SC −Ura medium (as described above, without agar) for overnight culture at 200 rpm at 30°C with shaking. Cells were washed 3 times with PBS and counted with a hemocytometer. Washed *C. albicans* cells were killed at a density of 1 × 10^8^ cells·ml^−1^ at 65°C for 30 min or by administration of 20 doses of UV at 100 mJ/cm^2^. Killed cells were washed twice more in PBS and plated on solid YPD to ensure nonviability. FITC staining of *C. albicans* was performed by suspending cells in 0.05 M carbonate-bicarbonate buffer (pH 9.6), adding FITC in dimethyl sulfoxide (DMSO) to a final concentration of 1 mg ml^−1^, and then washing the cells in PBS.

### Construction of plasmids and transfection into macrophages.

Murine cDNA for Rab5 was amplified from plasmid (OriGene, Rockville, MD) using proofreading KOD polymerase (Merck Millipore, Darmstadt, Germany) with the primers (Eurofins Genomics, Ebersberg, Germany) 5′ GCCGCCGAATTCCCATGGCTAATCGA 3′ and 5′ GAGCGGCCGGGATCCTAGTTACTACA 3′, generating flanking EcoRI and BamHI (Roche, Welwyn Garden City, United Kingdom) sites for cloning into pEGFP-C3 (Clontech, Mountain View, CA). Rab7 cDNA was digested from plasmid (OriGene) with SgfI and MluI (Roche) for cloning into pCMV-AN-tRFP (OriGene). Expression constructs were verified by sequencing (DNA Sequencing Services, University of Dundee, United Kingdom). RAW264.7 macrophages were transfected with pEGFP-Rab5, pCMV-AN-tRFP-Rab7, and commercially available pCMV-LifeAct-TagGFP2 (ibidi, Munich, Germany), using Lipofectamine LTX (Invitrogen) in antibiotic-free medium for 20 h prior to live-cell imaging.

### Phagocytosis assays and microscopy.

Standard phagocytosis assays were performed by combining a 3:1 ratio of yeast cells to adhered macrophages as described previously (62). Immediately prior to experiments, wells were replenished with fresh prewarmed supplemented RPMI, DMEM, or CO_2_-independent medium (Invitrogen) containing 1 µM LysoTracker Red DND-99 (Invitrogen) where required. Cells were incubated at 37°C (with 5% CO_2_ in experiments with RPMI or DMEM) for 2 to 18 h during image acquisition. For cathepsin experiments, Magic Red cathepsin reagent was added to wells according to the manufacturer’s instructions (ImmunoChemistry Technologies, Bloomington, MN). Phagocytosis assays were imaged using a DeltaVision Core microscope (Applied Precision, Issaquah, WA) or an UltraVIEW VoX spinning-disk microscope (Nikon, Surrey, United Kingdom) with Volocity software used for data analysis (Improvision, PerkinElmer, Coventry, United Kingdom). Localization of fluorescent reporters to individual phagosomes in live-cell movies was scored as positive upon the appearance of a halo of fluorescence, which was verified in representative examples as being above background by measuring a profile of intensity with Volocity line tool. Detection of lysed macrophages during phagocytosis assays in 96-well plates with 3.2 × 10^4^ macrophages and *C. albicans* cells at a multiplicity of infection (MOI) of 1:1, 3:1, or 10:1. YOYO-1 (Invitrogen) was added to supplemented DMEM at 0.1 µM prior to image acquisition with an Incucyte microscope (Essen Bioscience, Welwyn Garden City, United Kingdom). Macrophage actin was stained with rhodamine-phalloidin (Invitrogen) following phagocytosis of live FITC-stained *C. albicans* cells for 2 h. Cells were fixed in 2% paraformaldehyde for 10 min, permeabilized in 0.2% Triton X-100 in PBS for 5 min, and then washed. Fixation and permeabilization steps permitted macrophage but not fungal staining of actin by phalloidin. Calcofluor white (CFW) staining of fungal cell wall chitin was performed with 25 µg ml^−1^ CFW for 10 min and washing with PBS. A final concentration of rhodamine-phalloidin of 5-µg ml^−1^was used to stain cells for 20 min, before washing and capturing 3D images by spinning-disk microscopy. *C. albicans* was stained with soluble Dectin-1-Fc by washing cells from overnight cultures and then incubating them in supplemented DMEM at 37°C for 120 min to induce filamentation. Fungal particles were killed by UV or heat as required (described above). Dectin-1-Fc was incubated at 5 µg ml^−1^ with 2.5 × 10^6^ cells for 1 h on ice. Cells were washed three times with FACS buffer (0.5% bovine serum albumin [BSA], 5 mM EDTA, 2 mM Na azide in PBS) and resuspended in FACS buffer with 1/200 anti-human Fc-Alexa Fluor 488 with a 45-min incubation on ice. Cells were then washed 3 times with FACS buffer and fixed with 1% formaldehyde. All samples were examined by differential interference contrast (DIC) and fixed-exposure-time fluorescence microscopy using an Axioplan 2 microscope (Zeiss, Cambridge, United Kingdom). Images were recorded digitally by Openlab v 4.04 (Improvision). Transmission electron microscopy (TEM) was performed on cells from 2-h phagocytosis assays fixed with 1.5% glutaraldehyde at 4°C overnight and then dehydrated and embedded in an EMT-P automated tissue processor (Leica, Milton Keynes, United Kingdom), sectioned with a Leica UC7 ultramicrotome, contrast stained in a Leica AC20, and viewed with an UltraVUE transmission electron microscope (AMT, Woburn, MA).

### Assay for environmental alkalinization by *C. albicans*.

Medium to indicate alkalinization of the environment by *C. albicans* was prepared as described elsewhere (40). Liquid yeast nitrogen base (YNB) with 1% Casamino Acids and 20 mg liter^−1^ phenol red at pH 4 was inoculated with 1 × 10^6^ washed *C. albicans* yeast cells from overnight cultures. Alkalinization was indicated by a color change from yellow to red following 18 h of incubation at 37°C with shaking at 200 rpm. Solid YNB with 1% Casamino Acids, 2% agar, and 0.01% bromocresol green at pH 4 was inoculated with 1 × 10^3^ cells in a volume of 7 µl and incubated for 18 h at 37°C to generate colonies, with alkalinization indicated by a color change from green to blue.

### Flow cytometry.

PhagoFACS experiments were conducted using a method related to that already published (45) by isolating phagosomes from LTR or activated cathepsin B-stained J774.1 cells following standard phagocytosis assays for various time points with FITC-labeled *C. albicans* cells; noninternalized fungi were CFW counterstained as described above. Macrophages were ground in homogenization buffer (250 mM sucrose, 20 mM HEPES, 0.5 mM EGTA, 0.1% gelatin, 10 µM calpain inhibitor, 100 mM ATP [pH 7]) to liberate intact phagosomes for flow cytometry analysis of the percentage of phagosomes that were negative for CFW but positive for LTR or cathepsin B (gated by 405^−^, 488^+^, 561^+^) with an LSR II (BD, Oxford, United Kingdom). FACS determination of β-glucan exposure on the *C. albicans* cell surface was performed by inoculating YNB-glucose medium with cells from overnight cultures at an optical density (OD) of 0.2 with subsequent growth for 4 h at 30°C with shaking at 200 rpm. For each sample, 2.5 × 10^6^ cells were washed with FACS buffer and incubated with Dectin-1-Fc, as described above. Cells were washed with FACS buffer and then incubated with anti-human IgG-Alexa Fluor 488 for 40 min. Washed cells were fixed in 1% formaldehyde at 4°C. The unstained control comprised pooled samples and was incubated with FACS buffer alone during the first step and then treated with the secondary antibody to generate isotope control cells. Analysis was performed on a FACSCalibur (BD).

### Statistical analyses.

Data were analyzed by one-way analysis of variance (ANOVA) with Bonferonni’s *post hoc* comparisons, Mann-Whitney U test, or Student’s *t* test using GraphPad Prism 5.04 (GraphPad Software, Inc., La Jolla, CA).

## SUPPLEMENTAL MATERIAL

Figure S1 Representative image of *C. albicans* cells growing extracellularly and within macrophages. The scale is indicated by the bar, and dashed lines indicate the portion of hypha measured at that time point. (A) CAI4; (B) Δ*mnt1*/2 mutant. Download Figure S1, TIF file, 0.3 MB

Figure S2 FACS determination of mannan exposure on the cell surface of live, UV-killed, and heat-killed wild-type *C. albicans* yeast cells detected by ConA staining. (A) A representative histogram shows mean fluorescence intensities (blue, live; orange, UV killed; green, heat killed). (B) Mean fluorescence intensities of live, UV-killed, and heat-killed wild-type yeast cells. Download Figure S2, TIF file, 0.2 MB

Movie S1 Murine thioglycolate-elicited peritoneal macrophages phagocytosing live CAI4 cells over a 90-min period. Download Movie S1, MPG file, 9.4 MB

Movie S2 Murine thioglycolate-elicited peritoneal macrophages phagocytosing heat-killed CAI4 cells over a 90-min period. Download Movie S2, MPG file, 9.4 MB

Movie S3 J774.1 macrophages phagocytosing live CAI4 cells over a 5-h period. Download Movie S3, MPG file, 9.5 MB

Movie S4 J774.1 macrophages phagocytosing live Δ*mnt1*/*2* cells over a 5-h period. Download Movie S4, MPG file, 9.4 MB

Movie S5 RAW264.7 macrophages cotransfected to express eGFP-Rab5 and tRFP-Rab7, phagocytosing Δ*efg1* cells over a 32-min period. Download Movie S5, MPG file, 7 MB

Movie S6 Four-dimensional movie of RAW264.7 macrophages transfected with LifeAct to show actin dynamics of phagosomes containing CAI4 hyphae over a 42-min period. Download Movie S6, MPG file, 9.4 MB

Movie S7 Four-dimensional movie of RAW264.7 macrophages transfected with LifeAct to show actin dynamics of phagosomes containing Δ*mnt1*/*2* hyphae over a 20-min period. Download Movie S7, MPG file, 4.2 MB

Movie S8 Four-dimensional movie of RAW264.7 macrophages transfected with LifeAct to show actin dynamics of phagosomes containing Δ*efg1* cells over a 32-min period. Download Movie S8, MPG file, 9.4 MB
