# Supplementary figures and images for: Candida albicans Hypha Formation and Mannan Masking of β-Glucan Inhibit Macrophage Phagosome Maturation
Source: mBio. 2014 Dec 2;5(6):e01874-14. doi: 10.1128/mBio.01874-14 (PMC4324242; doi:10.1128/mBio.01874-14)

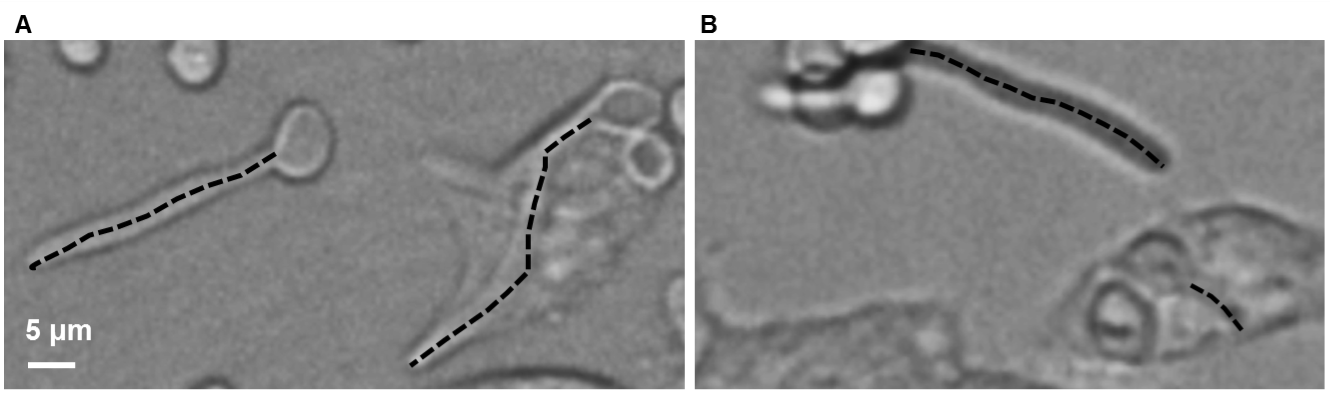

Supplement: Figure S1 — Representative image of C. albicans cells growing extracellularly and within macrophages. The scale is indicated by the bar, and dashed lines indicate the portion of hypha measured at that time point. (A) CAI4; (B) Δmnt1/2 mutant. Download [file mbo006142077sf1.tif]

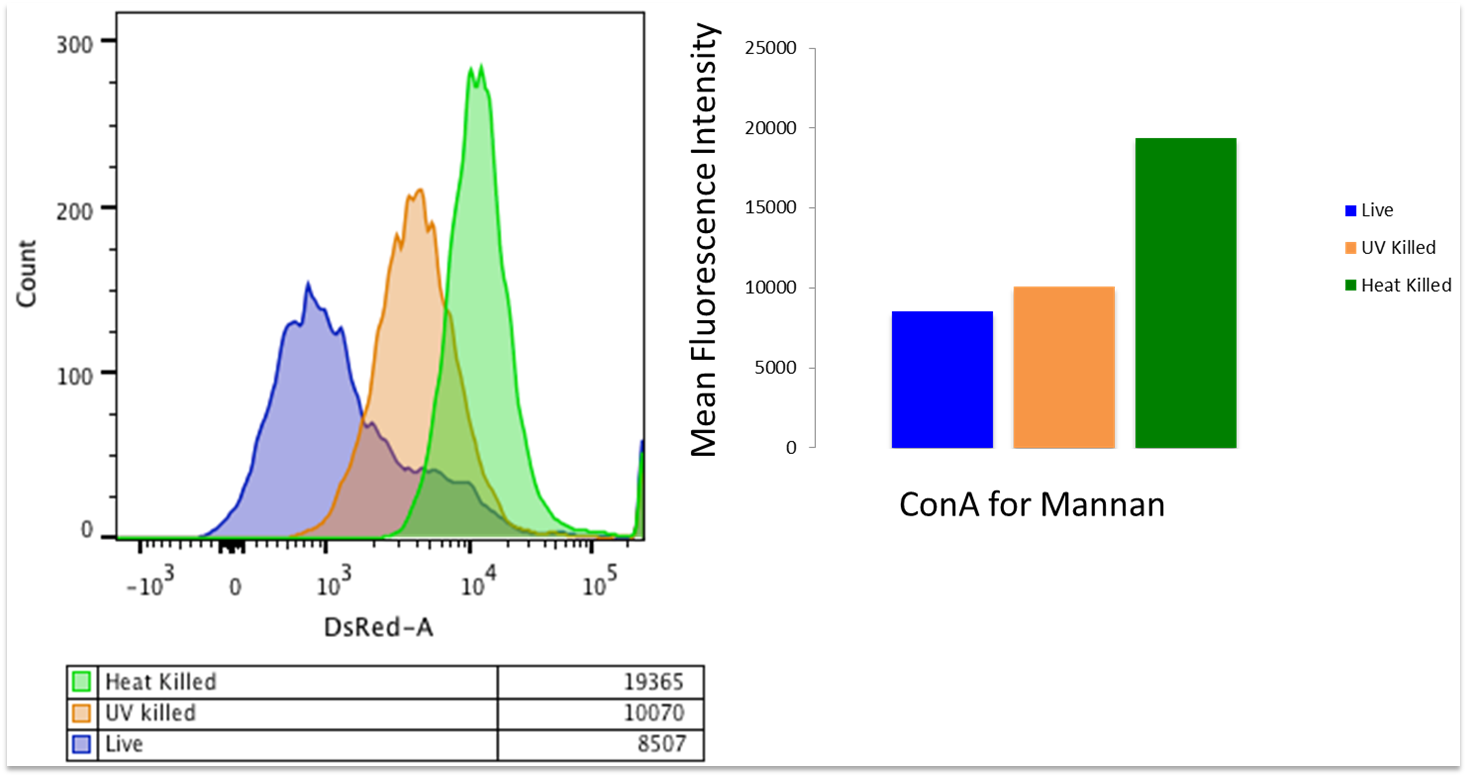

Supplement: Figure S2 — FACS determination of mannan exposure on the cell surface of live, UV-killed, and heat-killed wild-type C. albicans yeast cells detected by ConA staining. (A) A representative histogram shows mean fluorescence intensities (blue, live; orange, UV killed; green, heat killed). (B) Mean fluorescence intensities of live, UV-killed, and heat-killed wild-type yeast cells. Download [file mbo006142077sf2.tif]
